# Medium-chain fatty acid oxidation is independent of l-carnitine in liver and kidney but not in heart and skeletal muscle

**DOI:** 10.1152/ajpgi.00105.2023

**Published:** 2023-07-18

**Authors:** Andrea S. Pereyra, Kelsey L. McLaughlin, Katherine A. Buddo, Jessica M. Ellis

**Affiliations:** Department of Physiology and East Carolina Diabetes and Obesity Institute, Brody School of Medicine at East Carolina University, Greenville, North Carolina, United States

**Keywords:** acyl chain length, carnitine, fatty acid oxidation, mitochondria, medium-chain fatty acids

## Abstract

Medium-chain fatty acid (MCFA) consumption confers a wide range of health benefits that are highly distinct from long-chain fatty acids (LCFAs). A major difference between the metabolism of LCFAs compared with MCFAs is that mitochondrial LCFA oxidation depends on the carnitine shuttle, whereas MCFA mitochondrial oxidation is not. Although MCFAs are said to range from 6 to 14 carbons long based on physicochemical properties in vitro, the biological cut-off length of acyl chains that can bypass the carnitine shuttle in different mammalian tissues is unknown. To define the range of acyl chain length that can be oxidized in the mitochondria independent of carnitine, we determined the oxidative metabolism of free fatty acids (FFAs) from 6 to 18 carbons long in the liver, kidney, heart, and skeletal muscle. The liver oxidized FFAs 6 to 14 carbons long, whereas the kidney oxidized FFAs from 6 to 10 carbons in length. Heart and skeletal muscle were unable to oxidize FFAs of any chain length. These data show that while the liver and kidney can oxidize MCFAs in the free form, the heart and skeletal muscle require carnitine for the oxidative metabolism of MCFAs. Together these data demonstrate that MCFA oxidation independent of carnitine is tissue-specific.

**NEW & NOTEWORTHY** This work demonstrates that the traditional concept of mitochondrial medium-chain fatty acid oxidation as unregulated and independent of carnitine applies only to liver metabolism, and to kidney to a lesser extent, but not the heart or skeletal muscle. Thus, the benefits of dietary medium-chain fatty acids are set by liver metabolic activity and peripheral tissues are unlikely to receive direct benefits from medium-chain fatty acid metabolism, but rather metabolic byproducts of liver’s medium-chain oxidative metabolism.

## Graphical abstract

**Figure:**
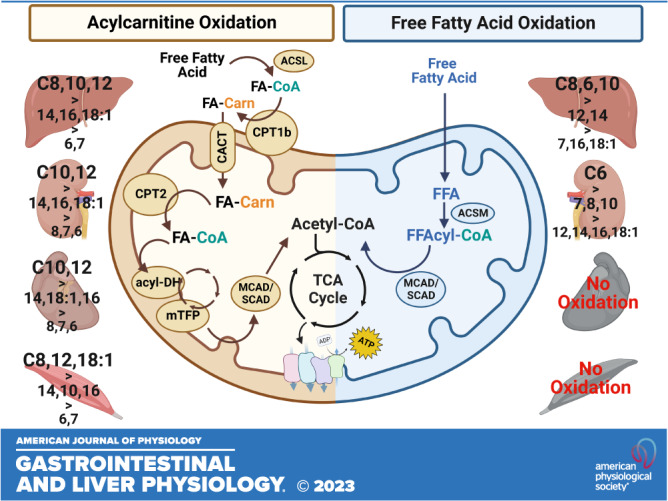

## INTRODUCTION

Dietary medium-chain fatty acids (MCFAs) are digested and oxidized distinctively from long-chain fatty acids (LCFAs). Expressly, upon ingestion, LCFAs within the enterocyte are incorporated into triglycerides and cholesterol esters and then packed as chylomicrons that travel through the lymphatic system and into the circulating blood, where chylomicron-derived LCFAs are available for uptake by target tissues. On the contrary, MCFAs are not packaged into lipoprotein particles in the enterocytes but released as free fatty acids (FFAs) into the portal vein for direct transit to the liver. Thus, although dietary LCFAs have a relatively low likelihood of being metabolized by the liver, dietary MCFAs are directed to this organ for metabolism.

At the cellular level, the energetic catabolism of LCFAs requires carnitine-mediated transport for entry into the mitochondria (1). On the contrary, MCFAs are believed to be oxidized independent of carnitine (2). Carnitine-dependent fatty acid oxidation is mediated by the sequential action of carnitine palmitoyltransferase 1 (CPT1), carnitine acylcarnitine translocase (CACT), and carnitine palmitoyltransferase 2 (CPT2), and it provides long-chain acyl-CoA to the β-oxidation spiral which produces acetyl-CoA, NADH, and FADH2. Mitochondrial acetyl-CoA can directly enter the citric acid cycle to generate more energy-producing metabolites, and in the liver, it can be used for ketone body synthesis. The carnitine shuttle exists mainly to regulate the fatty acid oxidative flux, preventing anabolism (fatty acid synthesis) and catabolism (fatty acid oxidation) from coinciding (3). The phenomenon that MCFAs are oxidized independent of carnitine bypassing the major regulated step that controls mitochondrial LCFA oxidation suggests rapid and continual flux of MCFA oxidation.

Despite these known distinctions between MCFAs and LCFAs, there is no standard to distinguish fatty acids with different acyl-chain lengths from a metabolic perspective. There is no consensus for the range of MCFA, with lower limits of 6 to 8 and upper limits of 10 to 14 carbons. Herein, the upper limits of MCFA classification were sought based on carnitine-free oxidative status in different mouse tissues. In the liver, FFAs up to 14 carbons long were readily oxidized independent of carnitine. The kidney, however, only oxidized FFAs up to 8 carbons in length. Interestingly, the heart and the skeletal muscle failed to oxidize any FFA from 6 to 18 carbons without carnitine. These results suggest a high level of tissue-specific fatty acid oxidative metabolism and that the ability of MCFAs to flux through fatty acid oxidation in a manner that bypasses CPT1 regulation is restricted to the liver and kidney, not occurring in the heart or skeletal muscle.

## METHODS

Mice of C57B6/J background were bred in-house with free access to water and standard chow (PicoLab 5053, Lab Diets) in pathogen-free housing under 12-h light-dark cycles. All procedures were approved by the Institutional Animal Care and Use Committee of East Carolina University (Assurance A3469-01).

Oroboros high-resolution respirometry was performed on fresh tissue homogenates from the heart, liver, kidney, or skeletal muscle (tibialis anterior + soleus muscles) of 4-mo-old male mice as described in previous reports (4–6). Specifically, tissues were homogenized in Mir05 buffer (MgCl_2_·6H_2_O 3 mM, K + MES 105 mM, taurine 20 mM, KH_2_PO_4_ 10 mM, HEPES 20 mM, d-sucrose 110 mM, and fatty acid-free BSA 1 g/L) at 20 wt/vol for liver and skeletal muscle and 40 wt/vol for kidney and heart using a Teflon on glass homogenizer. Respiration was measured in Mir05 media supplemented with EGTA (500 mM), creatine monohydrate (20 mM), and fatty acid-free BSA (0.1%) using 3 mg of tissue homogenate per milliliter of assay buffer. After endogenous homogenate respiration was steady, malate (2 mM; Sigma M1296) was added to maintain the tricarboxylic acid cycle, followed by ADP (2 mM) to stimulate respiration. We performed acyl-chain length-based titration assays to determine the maximum levels of substrate that would support respiration while avoiding membrane damage due to the detergent nature of some amphipathic lipids. We concluded that fatty acids between 6 and 8 carbons in length should be added at a maximum concentration of 0.2 mM. In comparison, acyl-chains of 10 carbons or longer should be added at a maximum concentration of 0.02 mM. Substrates used here were the following: hexanoate (Sigma 153745), hexanoyl-l-carnitine (Sigma 7439), heptanoate (Sigma W334812), heptanoyl-l-carnitine (Cayman Chemical 26551), octanoate (Sigma C5038) and octanoylcarnitine (Sigma 50892), decanoate (Sigma C1875), decanoyl-l-carnitine (Sigma 50637), laureate (Sigma W261408), lauroyl-l-carnitine (Sigma 39953), myristate (Sigma M3128), myristoyl-l-carnitine (Sigma 61367), palmitic acid (Sigma P0500), palmitoyl-l-carnitine mM (Sigma P1645), oleate (Sigma O7501) and oleoyl-l-carnitine (Sigma 19945). A total of 3–5 biological replicates were analyzed per tissue per acyl substrate.

Expression levels of CPT2 and CACT proteins in tissue homogenates were analyzed via immunoblot technique. Briefly, liver, kidney, heart, and skeletal muscle homogenates were heated at 95°C for 10 min in the presence of Laemmli sample buffer and β-mercaptoethanol and loaded in a 4%–20% precast polyacrylamide gel (Bio-Rad 4561096) at 20 µg per lane. Gels were subsequently transferred into a 0.45-µm nitrocellulose membrane, blocked with 5% milk in TBST buffer for 1 h, and incubated overnight at 4°C with primary antibodies (1:1,000) against carnitine palmitoyltransferase 2 (Millipore AB585), carnitine-acylcarnitine translocase (Proteintech 19363-1-AP), and heat shock protein 70 (Santa Cruz Antibodies 7298) as a loading control. Proteins were visualized using fluorescent dye-conjugated secondary antibodies (Li-Cor, 800CW or 680LT), and signal intensity analysis was performed using Image Studio suite (Li-Cor).

### Statistics

Data are presented as means ± SE. Statistical analysis and figures were generated using Excel or GraphPad Prism, v. 8.0.0 for Windows (GraphPad Software). Data were compared using one-way or two-way ANOVA followed by multiple comparison analysis. Student’s *t* test was used to compare respiration of FFA to the corresponding carnitine ester. Significance level was set at *P* < 0.05.

## RESULTS

### In the Liver, Carnitine-Free Respiration Spans from Hexanoate to Myristate

**Figure 1.**
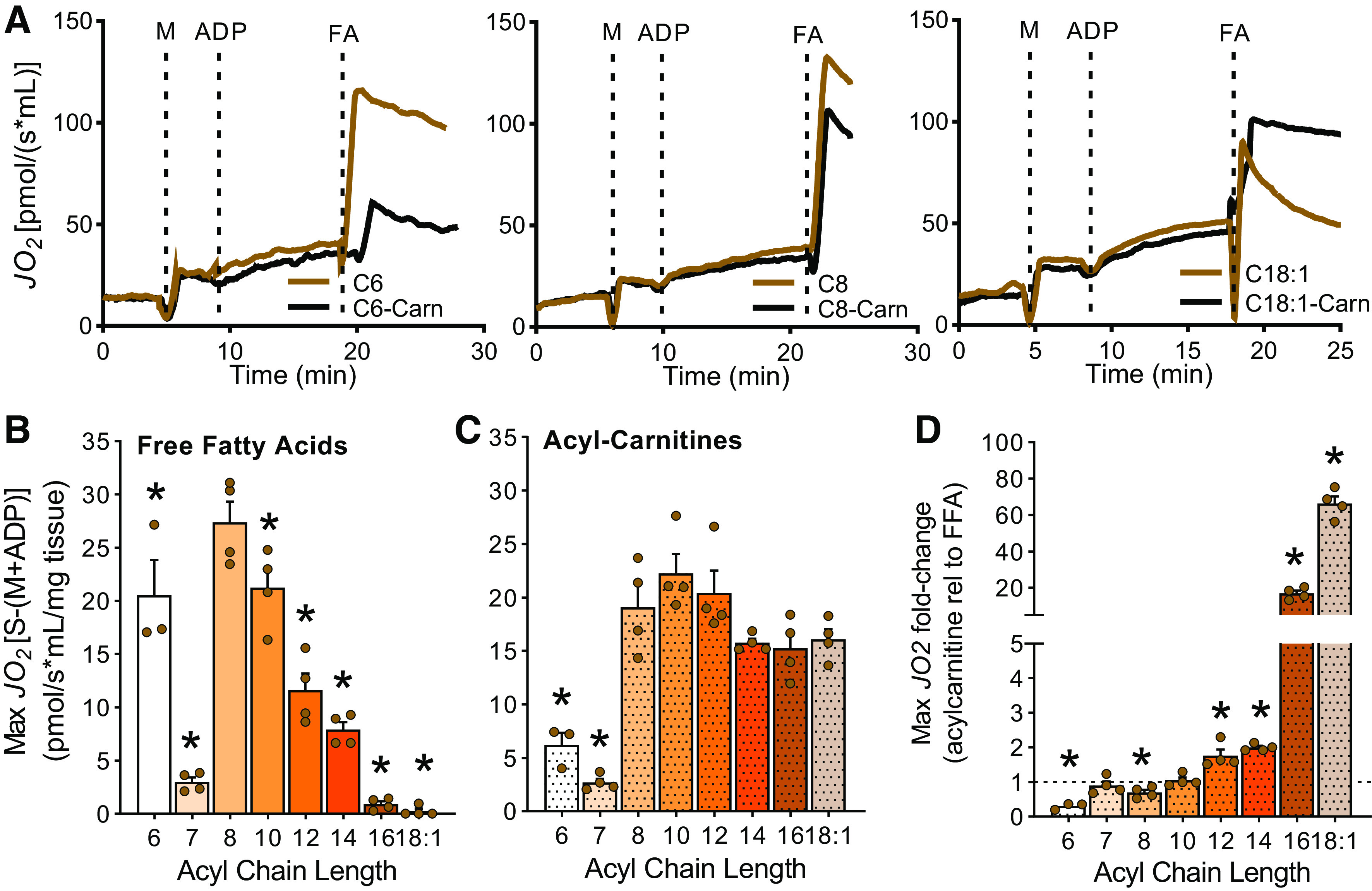
Liver fatty acid oxidation by chain length and carnitine dependence. *A*: representative traces of oxygen consumption rates (JO2) in liver homogenates over time following the addition of malate (M), ADP, and different fatty acids (FA) in either the free form or the carnitine ester (-Carn). *B*: quantitation of maximum respiratory rates in liver homogenate energized with free fatty acids (FFAs) between 6 and 18 carbons long, *n* = 3–4. *C*: quantitation of maximum respiratory rates in liver homogenate energized with acylcarnitines between 6 and 18 carbons long, *n* = 3–4. *D*: maximum respiration rate on acylcarnitine substrate relative to its FFA equivalent organized by acyl chain length, *n* = 3–4. Data are presented as means ± SE. Statistical analysis by one-way ANOVA relative to C8 (*B*) or to C8-Carn (*C*); for *D*, Student’s *t-*test was used to compared respiration of FFA with the corresponding carnitine ester. *P ≤ 0.05.

Dietary MCFAs are absorbed and delivered directly to the liver via the portal vein. To determine the chain length of fatty acids metabolized by the liver independent of l-carnitine, we measured the oxidation of FFAs between 6 and 18 carbons long. As a control, the oxidative flux of same-length carnitine ester was assessed in parallel. Oxygen consumption in liver homogenates was measured in realtime during the sequential addition of malate, ADP, and fatty acid (FA). Representative traces of mitochondrial respiration over time in liver homogenates energized with hexanoate (C6), octanoate (C8), and oleate (C18:1), as both free acids and carnitine esters, are depicted in Fig. 1*A.* Given that octanoate (C8) and octanoylcarnitine (C8-Carn) are commonly used to assess fatty acid-supported energy metabolism (7–10), we statistically compared each fatty acid oxidation rate with those of octanoate and octanoylcarnitine accordingly. Of the FFAs assayed, the liver achieved the highest respiratory rates when energized with octanoate (C8), decanoate (C10) and hexanoate (C6) respectively and independent of carnitine (Fig. 1*B*). Laureate (C12) and myristate (C14) were also readily oxidized but at ∼50% lower capacity relative to C8 (Fig. 1*B*). Heptanoate (C7) oxidation in liver occurred free of carnitine albeit at a low level, ∼90% less than octanoate (Fig. 1*B*). Liver had no respiratory capacity on palmitate (C16) or oleate (C18:1) (Fig. 1*B*). Maximum respiration levels for acylcarnitines ranging from 10 to 18 carbons long were similar to those for octanoylcarnitine (Fig. 1*C*). Respiration supported by hexanoylcarnitine (C6-Carn) and heptanoylcarnitine (C7-Carn), however, was 67 and 86% lower than on octanoylcarnitine, respectively (Fig. 1*C*). Comparison of the maximum respiration for the acylcarnitine ester relative to the equivalent FFA demonstrated that C6 and C8 are significantly more readily oxidized in the free form in the liver, whereas acylcarnitine esters from 12 to 18 carbons long were significantly more readily oxidized than the corresponding free forms (Fig. 1*D*). Together these data show that while acylcarnitines esters from 6 to 18 carbons long can be oxidized by the liver, only FFAs 6 to 14 carbons in length are oxidized independent of carnitine. Thus, the upper limit to distinguish MCFAs from LCFAs during oxidative metabolism is C14 in the liver.

### In the Kidney, Carnitine-Free Respiration Spans from C6 to C10

**Figure 2.**
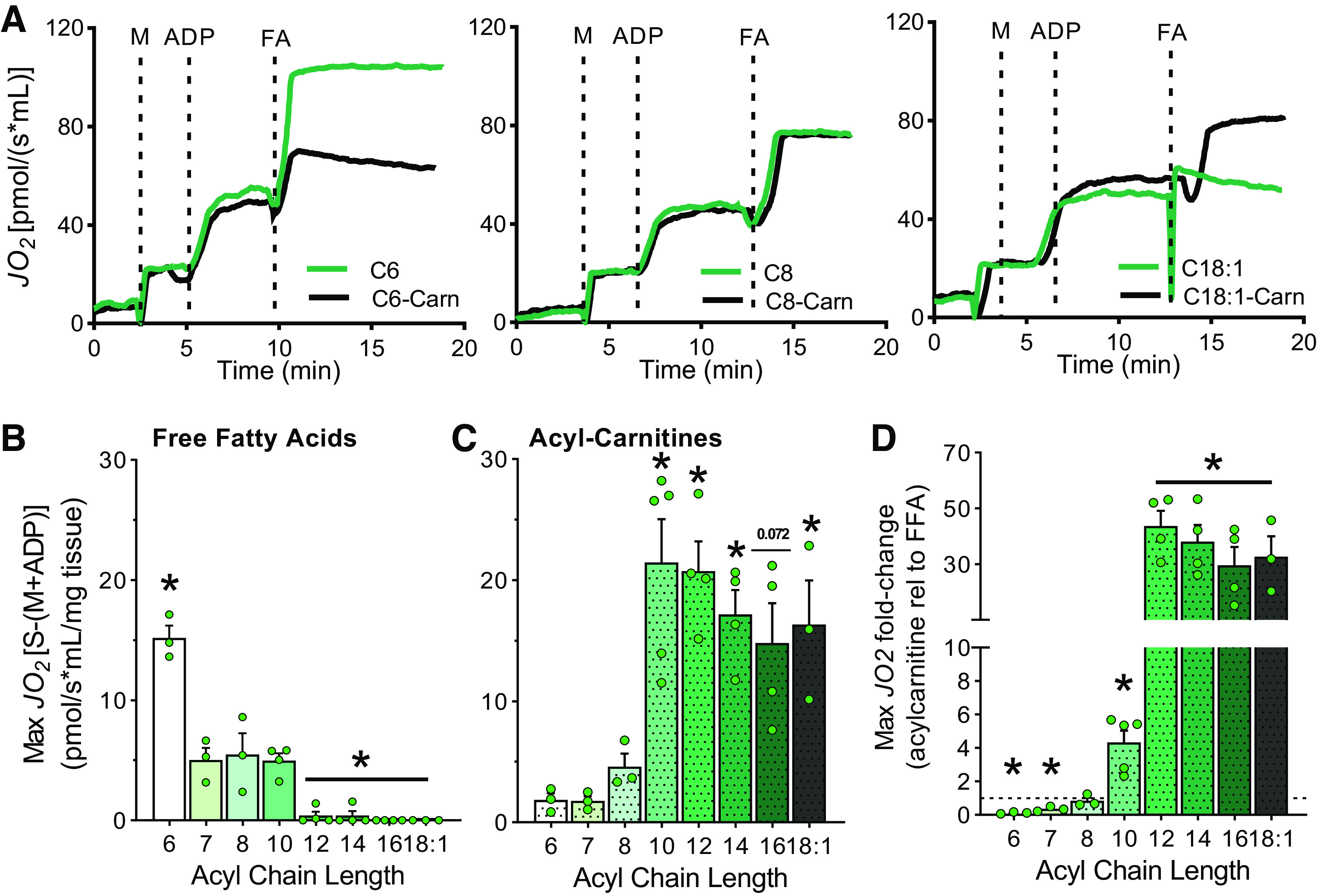
Kidney fatty acid oxidation by chain length and carnitine dependence. *A*: representative traces of oxygen consumption rates (JO_2_) in kidney homogenates over time following the addition of malate (M), ADP and different fatty acids (FA) in either the free or carnitine esters (carn). *B*: quantitation of maximum respiratory rates in kidney homogenate energized with free fatty acids (FFAs) between 6 and 18 carbons long, *n* = 3–4. *C*: quantitation of maximum respiratory rates in kidney homogenate energized with acylcarnitines between 6 and 18 carbons long, *n* = 3–4. *D*: maximum respiration rate on acylcarnitine substrate relative to its FFA equivalent organized by acyl chain length, *n* = 3–4. Data are presented as means ± SE. Statistical analysis by one-way ANOVA relative to C8 (*B*) or to C8-Carn (*C*); for *D*, Student’s *t*-test was used to compared respiration of FFA with the corresponding carnitine ester. *P ≤ 0.05.

Fatty acids are the preferred macronutrient to support kidney energy metabolism (11). Like the liver, the kidney supports metabolism in both an autonomous and systemic manner. It relies on fat oxidation to support its primary role of removing body waste and balancing internal fluids. To determine the kidney’s fatty acid oxidative capacity across chain lengths, oxidation of FFAs and carnitine esters was determined in kidney homogenates. Again, malate and ADP were added before the substrate to stimulate respiration. Representative mitochondrial respiration traces in kidney homogenates for C6, C8, and C18:1, in the free acid and the carnitine ester form, are depicted in Fig. 2*A.* Like the liver, the kidney was able to oxidize free C6, C7, C8, and C10 (Fig. 2*B*). However, unlike the liver, C7 and C8 were oxidized at similar capacity while C6 was oxidized at ∼250% higher rate in the kidney (Fig. 2*B*). Also contrary to liver, the kidney was not able to oxidize FFAs 12 and 14 carbons long (Fig. 2*B*). For the carnitine esters, C6-Carn, C7-Carn, and C8-Carn supported lower levels of respiration compared with longer chains (Fig. 2*C*). Oxidation of acylcarnitines from 10 to 18 carbons long was significantly higher relative to octanoylcarnitine (Fig. 2*C*). Comparing respiration on carnitine esters relative to FFAs in the kidney showed that respiration on free C6 and C7 was significantly higher than on the carnitine ester counterparts (Fig. 2*D*). On the contrary, rates on C8-Carn and free C8 were almost identical, and respiration on acylcarnitines 10 carbons and longer was significantly higher than on FFAs of the same length (Fig. 2*D*). These data demonstrate that the kidney can oxidize FFAs up to 10 carbons long, unlike the liver which can oxidize FFAs up to 14 carbons long. Furthermore, while the kidney can readily oxidize free hexanoate, it does not respire on C6-Carn. Thus, based on its FFA oxidation profile, the upper limit to distinguish MCFA from LCFA in the kidney is C10.

### Heart and Skeletal Muscle Require Carnitine to Oxidize Fatty Acids

**Figure 3.**
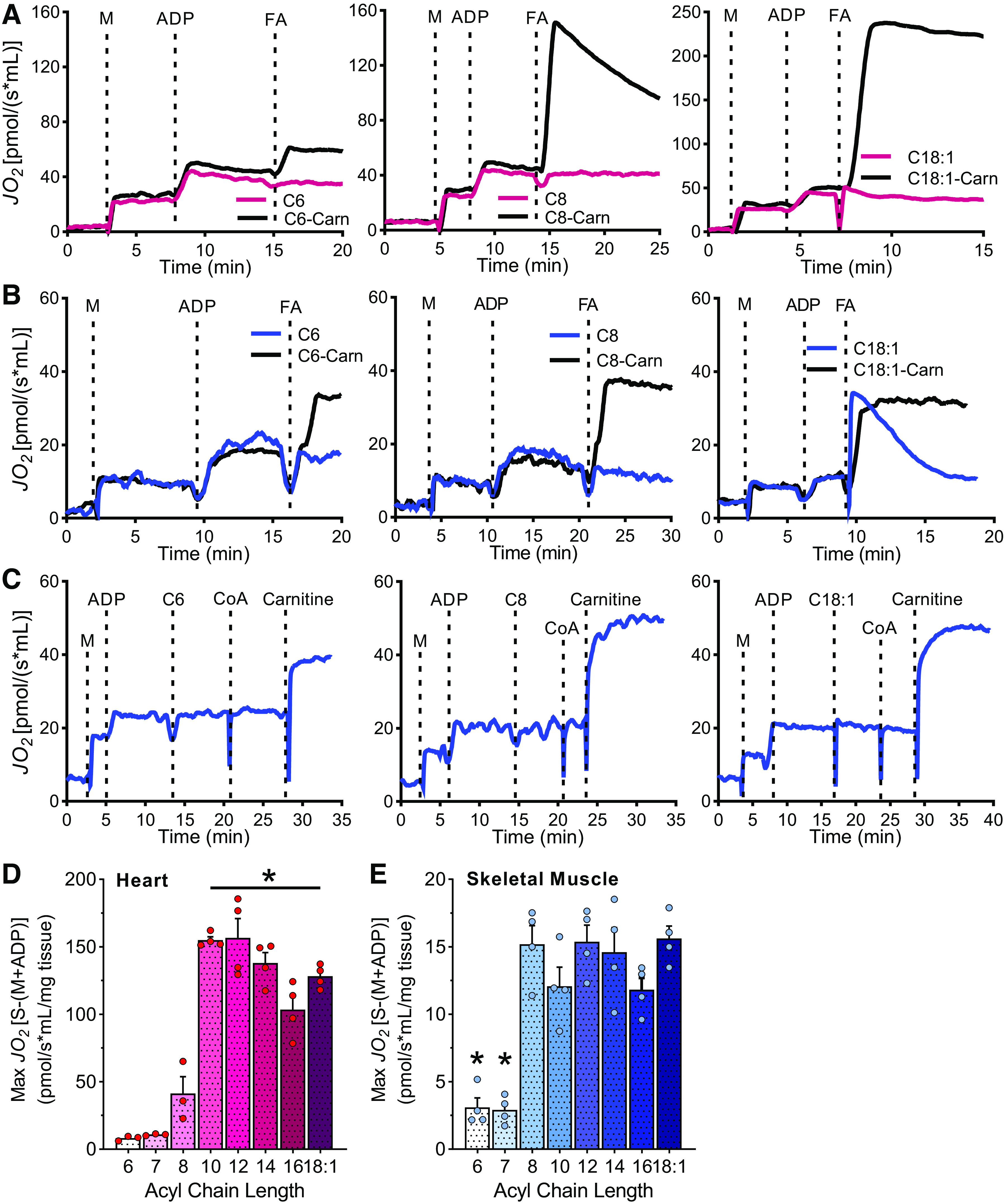
Heart and skeletal muscle fatty acid oxidation by chain length and carnitine dependence. Representative traces of oxygen consumption rates (JO_2_) in heart (*A*) and skeletal muscle homogenates (*B*) over time following the addition of malate (M), ADP and different fatty acids (FAs) in either the free or carnitine esters (carn). *C*: representative traces of oxygen consumption rates (JO_2_) in skeletal muscle homogenates over time following the addition of M, ADP, free FA, CoA and carnitine. Quantitation of maximum respiratory rates in heart (*D*) and skeletal muscle homogenate (*E*) energized with acylcarnitines between 6 and 18 carbons long, *n* = 3–4. Data presented as means ± SE. Statistical analysis by one-way ANOVA relative to C8-Carn. *P < 0.05.

Fatty acids are a critical energetic substrate during cardiac and skeletal muscle contraction. Because of the concept that MCFAs are oxidized in an unregulated manner, i.e., independent of CPT1, MCFA-enriched oils and supplements are often indicated in the treatment of metabolic myopathies, of disorders of carnitine synthesis and fatty acid oxidation, and to enhance exercise performance or treat diabetes (5, 12). Representative mitochondrial respiration traces in heart and skeletal muscle homogenates upon the addition of free and carnitine-bound C6, C8, and C18:1 are depicted in Fig. 3, *A* and *B*. Surprisingly, no respiration was detected in either heart or skeletal muscle when energized with FFAs of different acyl-chain lengths in the presence of malate and ADP (Fig. 3, *A* and *B*). To confirm that mitochondria were in good condition and able to oxidize free fatty acids in a carnitine-dependent manner, free coenzyme A (CoA) and free carnitine were added to skeletal muscle homogenates energized with free hexanoate, free octanoate, or free oleate demonstrating that the addition of CoA and carnitine allowed for robust oxidation of the FFAs (Fig. 3*C*).

When energized with acylcarnitines, the heart, and the skeletal muscle respired the highest on acyl-chains from 10 to 18 carbons long (Fig. 3, *A–E*). In skeletal muscle, octanoylcarnitine was oxidized at similar rates as longer acyl-carnitines (Fig. 3*E*). Compared with heart, the rates of acylcarnitine oxidation in skeletal muscle were ∼10-fold lower (Fig. 3, *D* and *E*). The oxidation rates for C6- and C7-Carn were low for both tissues and represented only 10% of the long-chain acylcarnitine-supported rate (Fig. 3, *D* and *E*).

These data demonstrate that heart and skeletal muscle cannot oxidize fatty acids of any chain length independent of carnitine. On the contrary, acylcarnitines of long acyl-chain lengths (C10 to C18) support high mitochondrial respiration in the heart and skeletal muscle, while shorter acylcarnitines (C6 to C8) are oxidized at lower rates. In summary, these data demonstrate that the concept of unregulated and carnitine-independent oxidation of MCFAs is a tissue-specific construct that does not apply to cardiac or skeletal muscles.

### The Capacity to Oxidize Free MCFAs for Energy Metabolism Is Tissue-Specific and Cannot be Generalized

**Figure 4.**
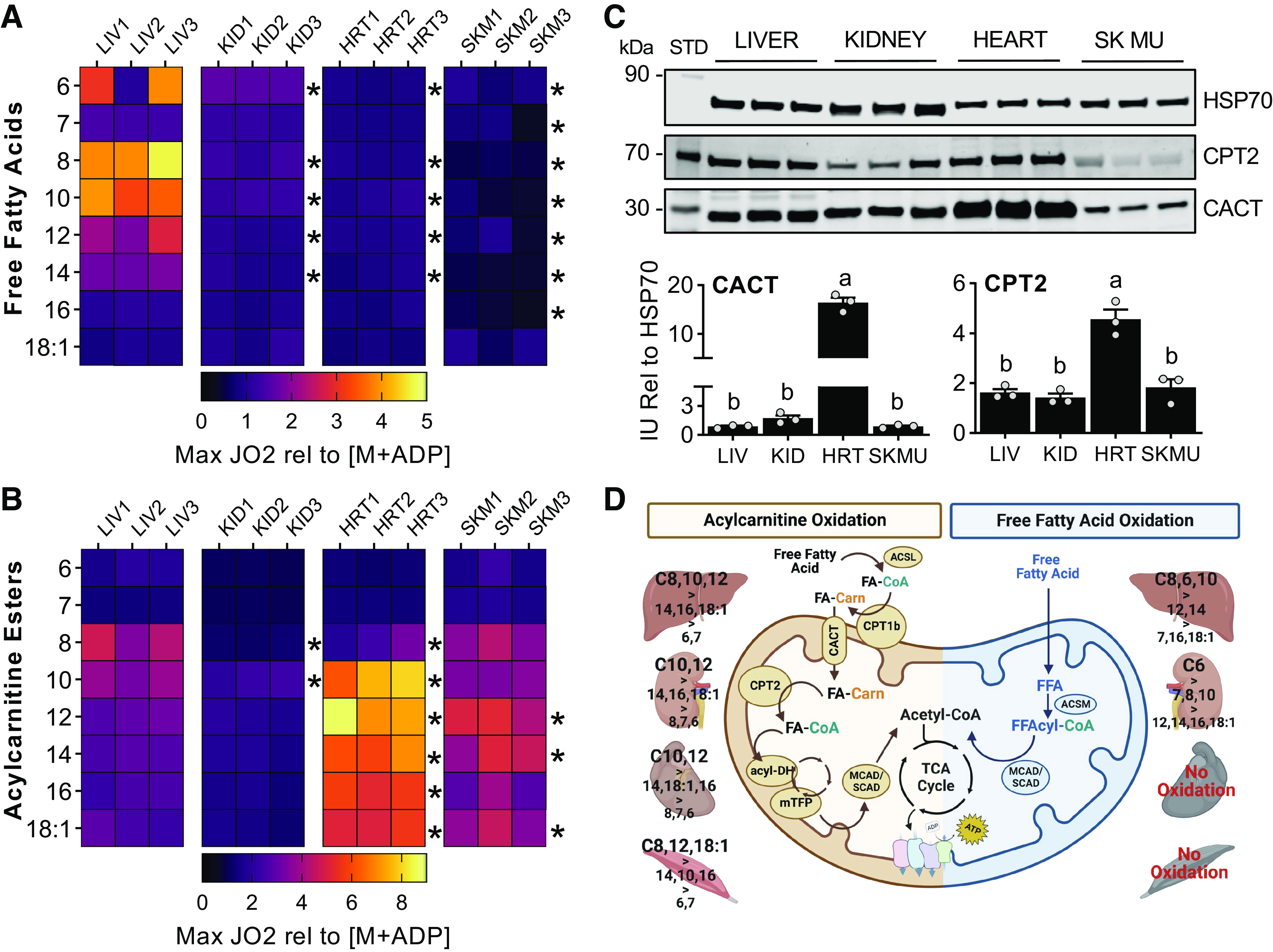
Intertissue comparison of fatty acid oxidation capability. Heatmap of Max Respiration rates (Max JO_2_) normalized to basal rates on malate+ADP ([M+ADP]) from liver (LIV), kidney (KID), heart (HRT), and skeletal muscle (SKM) energized with FFAs (*A*) or acylcarnitine esters (*B*) of different acyl-chain lengths, *n* = 3 per tissue. *C*: Western blot image and quantification of protein expression levels for carnitine palmitoyltransferase 2 (CPT2) and carnitine-acylcarnitine translocase (CACT) across tissues. Heat shock protein 70 (HSP70) was used as loading control. *D*: graphical summary of l-carnitine dependent and independent fatty acid oxidation across the different tissues. Statistical analysis by two-way ANOVA relative to liver (*A* and *B*) or by one-way ANOVA (*C*), *P ≤ 0.05. FA, fatty acid; ACSL, acyl-CoA synthetase long-chain; ACSM, acyl-CoA synthetase medium-chain; CPT1b, carnitine palmitoyltransferase 1 b; acyl-DH, acyl-CoA dehydrogenase; mTFP, mitochondrial trifunctional protein; SCAD, short-chain acyl-CoA dehydrogenase; MCAD, medium-chain acyl-CoA dehydrogenase.

To determine the extent that different tissues catabolize an array of fatty acids, we sought to compare fatty acid oxidative capability between the liver, kidney, heart, and skeletal muscle. The maximum respiratory rate of tissue homogenates energized with FFAs and the equivalent carnitine esters was made relative to basal respiration on malate + ADP. This normalization permits oxygen consumption analysis independent of differences in mitochondrial content. The liver oxidized free C6, C8, C10, C12, and C14 as evident by an increase from basal respiration by 2.6-, 4, 3.6-, 2.2-, and 1.8-fold, respectively (Fig. 4*A*). Comparing the kidney, heart, and skeletal muscle to the liver, respiration on these MCFAs, was significantly lower or nonexistent (Fig. 4*A*). No tissue was able to oxidize FFAs 16 or 18 carbons long (Fig. 4*A*).

When the fatty acids were administered as carnitine esters, the heart showed the highest oxidative capacity among all tissues, with respiration rates five- to seven-fold higher than basal for acylcarnitines between 10 and 18 carbons long (Fig. 4*B*). Octanoylcarnitine supported a significantly higher respiratory rate in the liver compared with heart and kidney (Fig. 4*B*). Like the heart, the skeletal muscle had high oxidation rates when energized with acylcarnitines from 8 to 18 carbons-long (Fig. 4*B*). Heart had highest rates of acylcarnitine oxidation of all tissues tested. To determine if these tissue-specific rates of oxidation could be due to differences in the abundance of the carnitine shuttle, we measured CACT and CPT2 protein expression levels in liver, kidney, heart, and skeletal muscle homogenates by immunoblot (Fig. 4*C*). Analysis showed that the heart had the highest expression of both, CACT and CPT2, while the liver, kidney, and skeletal muscle had levels similar to one another but significantly lower than the heart (Fig. 4*C*). These data suggest that the high rates of fatty acid respiration in the heart homogenates, relative to other tissues tested, are due to high levels of the fatty acid oxidative enzymatic machinery.

In summary, the liver is the only tissue capable of oxidizing FFAs up to 14 carbons long. At the same time, cardiac and skeletal muscles are entirely refractory to FFA-supported mitochondrial respiration (Fig. 4*D*). On the contrary, carnitine-dependent fatty acid oxidation is robust in the heart followed by the skeletal muscle, especially for acylcarnitines of 10 or more carbons-long. The liver, however, prefers shorter acylcarnitines of 8 and 10 carbons in length (Fig. 4*D*). The similar level of CACT and CPT2 expression between the liver, kidney, and skeletal muscle, does not correlate with the capacity to oxidize FFAs suggesting FFA oxidation occurs independent of the carnitine shuttle. Thus, these data together demonstrate that the ability to oxidize FFAs independent of carnitine for energy production is acyl-chain length and tissue dependent.

## DISCUSSION

MCFAs are present in the form of triglycerides in dietary sources such as milk and its derived products, in coconut, and in palm oil. Dietary MCFAs are digested and absorbed in the small intestine and exit the gut via the portal vein as FFAs for direct transit to the liver. Contrarily, the dietary LCFAs found in nearly all fat-rich foods are incorporated into triglycerides and packaged into chylomicron lipoprotein particles inside the enterocyte and are ultimately delivered to the general circulation by the lymphatic system. Thus, the liver is directly exposed to virtually all the dietary MCFAs in the free form but only to a relatively small degree of diet-derived LCFAs. The combination of access to dietary MCFAs with the unregulated nature of MCFA oxidation makes dietary MCFAs a prime source for liver acetyl-CoA production to promote ketogenesis. Indeed, dietary MCFAs are widely used to promote ketone body synthesis (13).

Ketone bodies are being increasingly recognized for conferring health benefits for numerous neurological, metabolic, and genetic diseases. In response, the consumption of foods rich in medium-chain fat and MCFA supplements is on the rise. However, it is not clear what the exact length cut-off is between MCFAs and LCFAs, and, more importantly, how this affects bioenergetic metabolism. For instance, coconut oils vary in the compositional distribution of MCFAs, making some of these oils solid and others liquid at room temperature. Specifically, longer-chain FAs have higher melting temperatures to thereby increase solidification, whereas shorter-chain MCFA, such as C8, have lower melting temperatures to promote the oil-like nature of products. Herein, the oxidative metabolism for a wide range of fatty acyl chains was determined for liver, kidney, heart, and skeletal muscle to determine the oxidative capacity of FAs across chain lengths. We found that the liver can oxidize FFAs up to 14 carbons in length, suggesting that the concept of carnitine-independent, unregulated, MCFA oxidation extends up to 14 carbons in the liver. This phenomenon did not replicate in kidney, heart, or skeletal muscle. Thus overall, the liver has direct access to dietary MCFAs, has high rates of MCFAs oxidation, can oxidize MCFA independent of carnitine, and is the tissue where ketone bodies are generated. These data suggest that a vast majority of dietary MCFAs flux through liver metabolism to generate energy and/or ketone bodies.

MCFAs, particularly C8, can also be generated intracellularly as a product of peroxisomal fatty acid oxidation (14). Peroxisomes oxidize very-long fatty acids from 20 to 24 carbons in length as well as branched-chain fatty acids. The primary end product of peroxisomal oxidation is C8-CoA. C8-CoA is converted to C8-Carn by the peroxisomal enzyme carnitine *O*-octanoylcarnitine transferase for subsequent export and uptake by the mitochondria for further catabolism. Thus, the completeness of peroxisomal fatty acid oxidation depends on mitochondrial oxidation. Tissues with high rates of peroxisomal fatty acid oxidation, such as heart and skeletal muscle, likely expose mitochondria to high levels of C8-Carn. We previously demonstrated that muscle can oxidize C8-Carn without the mitochondrial matrix enzyme CPT2. Conceivably, peroxisomal fatty acid oxidation occurs in a manner that bypasses the regulatory nodes that restrict mitochondrial LCFA oxidation. Thus, tissues such as muscle may have adapted to use C8-Carn as a mitochondrial substrate, independent of the acylcarnitine shuttle to allow complete oxidation of peroxisomal-derived substrates. Thereby, active peroxisomal oxidative metabolism is not subjected to bottlenecking of C8-Carn by mitochondrial-regulatory processes. This concept is supported by unphased rates of C8-Carn oxidation in the absence of CPT2, a critical component of the long-chain mitochondrial fatty acid oxidation pathway.

In addition to peroxisomes generating C8-Carn, MCFA supplements typically have a high abundance of free C8. Work presented here demonstrates that C8, and its carnitine ester, C8-Carn are readily oxidized in the liver but not in kidney, heart, or skeletal muscle which prefer longer chain acylcarnitines to support respiration. This intertissue difference in C8 and C8-Carn oxidative metabolism in favor of liver might be due, in part, to the specialized hepatic metabolism for lipoic acid synthesis which utilizes C8 acyl chains as substrate (15). Another MCFA of clinical relevance is heptanoate (C7). Its triglyceride form, triheptanoin, is used in the treatment of long-chain fatty acid oxidation disorders (16). Heptanoate is metabolized to acetyl-CoA and propionyl-CoA, an anaplerotic intermediate that feeds the citric acid cycle to promote metabolic flux. Thus, although we found the rates of heptanoate oxidation were fairly low compared with octanoate, this might be the result of our assay, which measures complete oxidation, not necessarily reflecting anaplerosis. The anaplerotic nature of heptanoate metabolism might be better reflected using label-based metabolic tracing studies.

Another critical point our data highlights is the complete absence of mitochondrial respiration in the heart and skeletal muscles when energized with FFAs of any length. This phenomenon is often underestimated when considering the real therapeutic effects of MCFAs in muscle tissues affected by long-chain fatty acid oxidation disorders, which seem more likely mediated by the hepatic supply of MCFA-derived ketone bodies rather than by MCFAs directly acting as a carbon source in muscle.

We have previously shown that a stark difference between liver and muscle is the expression of medium-chain acyl-CoA synthetases (ACSMs), which activate free MCFAs into MCFA-CoAs within the mitochondria potentially to facilitate fatty acid oxidation (5). The high abundance of ACSMs in the liver and lack of expression in muscles may allow the liver to oxidize FFAs, but not the heart and skeletal muscle. Here, we evaluated if differential expression of critical elements of the carnitine shuttle such as CACT and CPT2 could contribute to the tissue-specific fatty acid oxidative profile. We conclude that the significantly higher levels of CACT and CPT2 in the heart compared with the liver, kidney, and skeletal muscle agree with the robust mitochondrial respiration on acylcarnitines ≥10 carbons long observed in the heart. Furthermore, the fact that liver, kidney, and skeletal muscle present with similar levels of CACT and CPT2 reinforces our idea that the carnitine shuttle is not likely involved in the oxidation of FFAs since skeletal muscle does not respire when energized with FFAs of any acyl-chain length while liver and kidney do.

Overall, our findings demonstrate that the traditional concept of mitochondrial MCFA oxidation being unregulated and independent of carnitine applies only to liver metabolism, and to a lesser extent, to the kidney. Furthermore, we propose that the biologically relevant acyl length cut-off between MCFAs and LCFAs is tissue-specific and cannot be generalized.

## GRANTS

This work was supported by the National Institutes of Health Grant R01-DK125812 (to J.M.E.).

## DISCLAIMERS

The content is solely the responsibility of the authors and does not necessarily represent the official views of the National Institutes of Health.

## DISCLOSURES

No conflicts of interest, financial or otherwise, are declared by the authors.

## AUTHOR CONTRIBUTIONS

A.S.P. and J.M.E. conceived and designed research; A.S.P., K.L.M., and K.A.B. performed experiments; A.S.P. analyzed data; A.S.P. interpreted results of experiments; A.S.P. prepared figures; A.S.P. and J.M.E. drafted manuscript; A.S.P. and J.M.E. edited and revised manuscript; A.S.P. and J.M.E. approved final version of manuscript.
